# Comparing the Predictive Ability of Prognostic Models in Ischemic Stroke; Derivation, Validation, and Discrimination Beyond the ROC Curve

**DOI:** 10.3389/fneur.2014.00009

**Published:** 2014-01-27

**Authors:** Alireza Esteghamati, Nima Hafezi-Nejad, Sara Sheikhbahaei, Behnam Heidari, Ali Zandieh, Vahid Eslami

**Affiliations:** ^1^Endocrinology and Metabolism Research Center (EMRC), Vali-Asr Hospital, School of Medicine, Tehran University of Medical Sciences, Tehran, Iran

**Keywords:** prediction models, decision analysis, stroke, prognosis, discrimination

A number of new studies have introduced a different risk score in contrast to National Institute of Health Stroke Scale (NIHSS) to predict prognosis in ischemic stroke (1, 2). Other recent studies have evaluated NIHSS and compared traditionally established risk scores, with newly modified models (3–8). New modeling can ease the access of scaling systems, develop a better educational background, and reveal the main common basis of the more complex models. Though the idea is clever, we’d like to highlight three educational concerns regarding the handling of the future studies on NIHSS, stroke scaling, and relative comparisons.

## First: NIHSS vs. Stroke Impact Scale

First of all, the NIHSS was not intended as a predictor of outcome from stroke. Rather, it was intended to standardize the degree of neurological deficit in acute stroke so that treatments for acute stroke could be compared based upon how severe the stroke was. In recent years, many authors have used the scale as a predictor but that was not the intent and hence there are problems with it in this regard. In contrast, other measures like the Stroke Impact Scale (SIS) were designed for prognostic purposes. We suggest using SIS, which covers eight dimensions and a composite disability score, for assessing the outcome in acute stroke (9). SIS has shown to be a feasible, reliable, valid, and sensitive scale (9). Even proxies can provide valid information for assessing the stroke outcome by applying SIS (10). This is a great advantage, especially when using SIS for research purposes.

## Second: Assessing a Sensitive Method and Claiming the Clinical Utility

This is how the story goes on in many surveys: they typically derive a scoring system from their cohort of ischemic stroke patients and compare it to a standard established model like NIHSS. The comparison of the new model with traditional NIHSS may reveal modest though non-significant decrement, and the interpretation admits the applicability of the newer method. However, when Receiver Operating Curve (ROC) is the main applied method, we should consider further analysis. Recent studies have shown that C-statistics is not sensitive to show discrimination of an additive model (11). C-statistics (exp: ROC curve and corresponding Area Under the Curve, AUC) loses its ability in detecting the discriminatory difference, especially with regard to outcomes’ prevalence (12, 13). As in most cases, when the baseline predictive ability is considerable (AUC ≳ 0.80), incremental AUC wouldn’t go beyond minor changes. Despite large difference in discriminatory power of two comparing models, AUC may show minimal decline and thus fail to reveal the prediction superiority. Applying newer methods, which are more sensitive is the plausible manner we should look for (14). As a simple example, Integrated Discriminatory Improvement (IDI) would be a suitable choice, especially when the authors’ objective is to choose a simpler method to be as powerful as the traditional NIHSS by using discriminant analysis. IDI is calculated by comparing the discrimination slopes of the two models (13–15). Absolute IDI’s interpretation remains to be understood. However, as stated by Pencina et al. relative IDI (rIDI, equaling IDI divided by the traditional model’s slope) has an “intuitive” definition (15). rIDI can show the portion of the traditional model, which can be explained by a new method. One can simply calculate the discrimination slopes, their difference (IDI), and the percentage of improvement (or failure) from traditional NIHSS to a new model. This rIDI matches the percentage of the predictive prognostic value of NIHSS, which can be explained by the variables in the new model. In the other word, we can explain the percentage of improvement we gain (or lose) by summarizing the complex NIHSS to a new pointing system. This is far more applicable. Recent studies have claimed that the rIDI can assess the “Clinical utility” (15) of the model as well.

Comparisons with NIHSS can be more clarified by taking the time to event (TTE) into account. TTE is an important component of prediction models, specifically in case of stroke (16). TTE seems to be the next missing point in most of such cohorts. IDI can also be estimated using the TTE values of the studied cases (14, 15).

We exemplify the usage of discriminant slopes, IDI, rIDI and their superiority in comparison with C-statistics, and ROC curve analysis, in a series of 117 consecutively referred patients to our private clinic. All patients had been finally diagnosed as having an ischemic stroke event. Neither of them had previous history of ischemic events, nor was receiving treatments prior to the event. NIHSS items and the incidence of mortality were recorded. Here we compare two short NIHSS (sNIHSS) scores introduced by Tirschwell et al. for utilization in pre-hospital settings (1). Eight of the NIHSS items were selected as follow: right leg, left leg, gaze, visual fields, language, level of consciousness, facial palsy, and dysarthria. The two introduced models were defined as sNIHSS-8 and sNIHSS-5, including the first eight and the first five, of the afore-mentioned items, respectively. In our simulation we used binary logistic regression analysis with mortality as the dependent variable (0 or 1) and each model’s items as the covariates. Probabilities of the sNIHSS-8 and sNIHSS-5 were saved and used for the comparison of the two models. In ROC curve analysis, the AUC was 0.943 (0.882–1.000) and 0.922 (0.816–1.000) for sNIHSS-8 and sNIHSS-5, respectively. As you see, AUC results were almost similar with no statistical significant difference among the two models. Next, we calculated the discrimination slope, IDI and rIDI for the two models. By shortening the sNIHSS-8 to the sNIHSS-5, the discrimination slope reduced from 0.62 to 0.55, IDI was reduced by −0.07 and rIDI was calculated as ~13%. Practically, this means that we lost up to 13% of our predictor power; while C-statistics failed to show any decrement, which was due to the large predictor power of the baseline model.

As we explained and exemplified, ROC curve analyses (and C-statistics in general) are not sensitive to change when the baseline model has already a large power of prediction (14). This is what almost always happens with validated scoring systems. To detect smaller changes in the model, we suggested using a more sensitive effect measure estimator, including discrimination slope and IDI. By using them, we can detect smaller changes and come up with a more realistic estimation of change. Besides, for every obtained effect size, we can increase our precision by using techniques that provide us with a more definitive result; like having narrower confidence intervals for testing a hypothesis. In ROC curve analyses, obtaining a precise standard error will become critical when especially binormal assumptions about the latent frequency distributions of test results are not met (17). Re-sampling methods can aid us in attaching a distribution-independent standard error, to a point estimate. Jackknife [by Tukey (18)] and bootstrapping [by Efron (19)] are the two most famous re-sampling methods (20). They act as companions to a sensitive effect size. In fact, Jackknife and bootstrapping are measures of precision, whereas, sensitive effect estimators are measures of accuracy. While the former deals with reproducibility, the latter deals with reality.

## Third: Derivation vs. Validation Cohort

When authors derive a scoring system from their cohort of stroke patient, statistical analysis results in the best fitted predictive model using the new method’s variables in their cohort. The cohort which gives born to the model is so called as the “Derivation” or “Construction” cohort. Similar to several previous models in different fields of medicine (21), and specifically in predicting cardiovascular events (22), one would expect the model to be tested, compared or so called as “Validated” in a different, separate, and independent cohort. The predictive ability of the new model in the sample it has been derived from (and thus fits by definition) is not indicative. Further evaluations on different samples are always needed to admit the validity of a new model.

Finally, we conclude that using a validation cohort accompanied by acquiring more sensitive measures can reveal the predictive value of the short scoring systems and newer methods in comparison to NIHSS or other established scales.
